# PollenDetect: An Open-Source Pollen Viability Status Recognition System Based on Deep Learning Neural Networks

**DOI:** 10.3390/ijms232113469

**Published:** 2022-11-03

**Authors:** Zhihao Tan, Jing Yang, Qingyuan Li, Fengxiang Su, Tianxu Yang, Weiran Wang, Alifu Aierxi, Xianlong Zhang, Wanneng Yang, Jie Kong, Ling Min

**Affiliations:** 1 National Key Laboratory of Crop Genetic Improvement, Huazhong Agricultural University, Wuhan 430070, China; 2 Institute of Economic Crops, Xinjiang Academy of Agricultural Sciences, Urumchi 830091, China; 3 Forestry and Fruit Tree Research Institute, Wuhan Academy of Agricultural Sciences, Wuhan 430075, China

**Keywords:** computer vision, deep learning, high temperature stress, open-source, pollen viability

## Abstract

Pollen grains, the male gametophytes for reproduction in higher plants, are vulnerable to various stresses that lead to loss of viability and eventually crop yield. A conventional method for assessing pollen viability is manual counting after staining, which is laborious and hinders high-throughput screening. We developed an automatic detection tool (PollenDetect) to distinguish viable and nonviable pollen based on the YOLOv5 neural network, which is adjusted to adapt to the small target detection task. Compared with manual work, PollenDetect significantly reduced detection time (from approximately 3 min to 1 s for each image). Meanwhile, PollenDetect can maintain high detection accuracy. When PollenDetect was tested on cotton pollen viability, 99% accuracy was achieved. Furthermore, the results obtained using PollenDetect show that high temperature weakened cotton pollen viability, which is highly similar to the pollen viability results obtained using 2,3,5-triphenyltetrazolium formazan quantification. PollenDetect is an open-source software that can be further trained to count different types of pollen for research purposes. Thus, PollenDetect is a rapid and accurate system for recognizing pollen viability status, and is important for screening stress-resistant crop varieties for the identification of pollen viability and stress resistance genes during genetic breeding research.

## 1. Introduction

Pollen is an important research subject in botany, climatology, ecology, and other fields. As the male gametophyte in the sexual reproductive stage of plants, pollen plays an important role throughout reproductive development in plants. The development of pollen, the combination of male and female gametophytes, and the germination and growth of the pollen tube are important processes that determine the reproductive quality of plants. Pollen is subjected to a variety of environmental stresses during its development, including, but not limited to, low temperature, high temperature, radiation, and drought. Some of these factors act directly on the pollen itself, while some act on stamens and even on the whole plant. For example, the processes of stamen development, pollen germination on the stigma, and pollen tube growth in cotton are very sensitive to temperature, and high temperature will lead to a decrease in pollen viability and hinder pollen fertilization and the development of cotton bolls, resulting in a decline in cotton yield [1,2,3]. Botanists have worked diligently to identify genes that affect pollen viability, and have combined gene editing technologies to improve the resistance of plant pollen to adverse stresses, expand planting areas, and increase crop yield [4,5].

Researchers have also explored the best observation and statistical methods for assessing pollen development. The traditional method for identifying the viability of pollen is manual observation through microscopy after staining, including bright- and darkfield microscopy, which can be combined with different pollen preparation methods, such as fresh pollen staining or acetolysis [6]; the latter can also be used for fossil pollen [7]. Thus, botanists who need to obtain large-scale (e.g., population-scale) data on pollen phenotypic status would need to invest a great deal of effort in much repetitive work to determine the phenotype of pollen by manual observation. It is obvious that the traditional pollen viability detection method is easily subject to the influence of tedious operation steps and subjective factors of manual operation. To improve the efficiency of genetics and breeding research, a fast pollen viability identification method with high accuracy is needed by researchers. With technological and equipment development, researchers have made great progress in automatic analysis and computer-aided observation of pollen, and many new pollen analysis methods have emerged, such as molecular methods: metabarcoding [8], genome skimming [9], and chemotaxonomy [10]. Image detection technology based on deep learning convolutional neural networks (CNNs) and related machine learning technology, which have the advantages of high efficiency, accuracy, and rapidness, has the potential to advance pollen analysis.

To date, researchers have made some exciting achievements in agriculture through deep learning techniques combined with molecular biology methods [11]. For example, technicians developed the MicroScan system, which has been used to detect the pollen developmental stages of different eggplant varieties, and the detection accuracy has reached 86.3% [12]. By combining this system with the high-throughput genotyping platform of single primer enrichment technology, haploid and diploid plants were identified with a high recognition rate by flow cytometry. Moreover, to detect apple growth period in real time and estimate apple yield, the DenseNet method was used to improve the YOLOv3 model to overcome the limitation of detection being applicable at specific growth stages, thus realizing the high-throughput and rapid detection of apples at different growth stages with the same model [13]. To improve tea picking efficiency, the researchers trained a model through the YOLOv3 model. It can automatically locate the picking point of tea leaves, and the successful picking rate of tea leaves was 80% [14]. In order to accurately predict the orange yield of an orchard, researchers trained the YOLO model to detect oranges under different lighting fields and constructed a yield estimation method. The spatial distribution error of the final fruit yield was only 9.19% [15]. At the same time, nondestructive testing of mango fruits is also emerging with the help of deep learning models [16]. At present, pollen classification techniques based on deep learning in plant science are booming, and can distinguish the pollen of 30 different crops in the same data set [17]. However, the detection and classification of pollen viability using deep learning technology have not been considered in detail.

In this study, we combined the PyTorch deep-learning-framework-based YOLOv5 algorithm and computer vision to develop a high-throughput automatic pollen viability recognition tool (PollenDetect). This tool can be used to automatically detect a large number of pollen grains from images for classification, counting, and pollen viability recording. We obtained a recognition accuracy of 99% when applying PollenDetect to a cotton pollen data set. The robustness of this tool for pollen viability detection in different plants (*Arabidopsis*, corn, camellia, and azalea) was evaluated. Finally, a visual interface for PollenDetect was created and released on GitHub (https://github.com/Tanzhihao1998/Identification-of-pollen-activity.git, accessed on 1 April 2022), which can be used for pollen viability detection and counting on computers with only a CPU by botanists without a computer science background.

## 2. Results

### 2.1. Pollen Viability Detection Algorithm Design and Training

**Figure 1:**
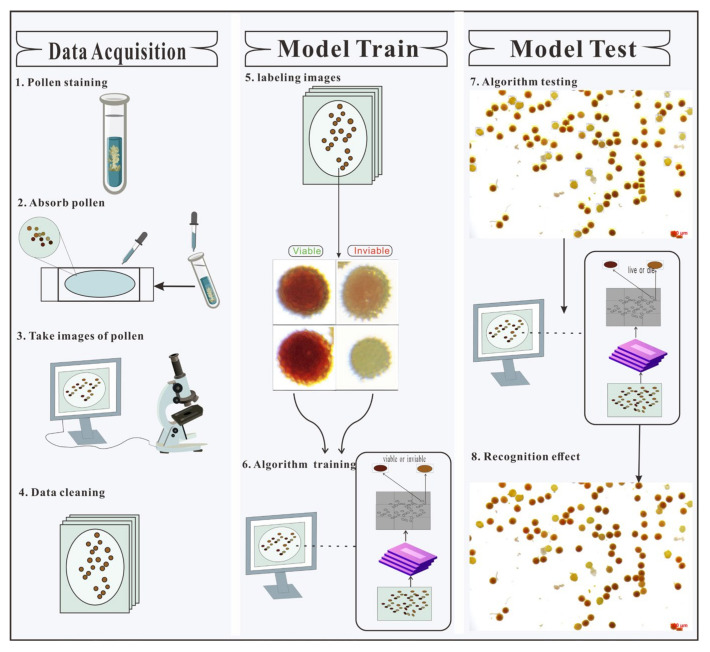
Pipeline for algorithm development. (1) The pollen was placed in TTC solution for 1 h. (2) The supernatant was absorbed and placed on a glass slide. (3) The cotton pollen image was taken under a binocular microscope with a camera. (4) The captured pollen image was cleaned and the unclear images were removed. (5) The cleaned pollen image was marked with image marking software to distinguish viable and nonviable pollen. (6) Pollen images and tagging files were fed into the deep learning model for training. (7) A set of new images was sent to the trained deep learning model for detection to evaluate model performance and select the best model. (8) Each pollen grain was marked with its category in the detected image.

We used YOLOv5, Faster R-CNN (PaddlePaddle 1.7), and YOLOv3 deep learning models to construct three pollen viability detection systems that can detect pollen viability by computer instead of manual work. The construction process of each system is shown in Figure 1.

The viable pollen was dark red and reddish brown after 2,3,5-triphenyl tetrazolium chloride (TTC) staining, while the nonviable pollen was light yellow or gray. Thus, we were able to distinguish the viable and nonviable pollen by color characteristics. Some scholars have shown that transfer training can enable the underlying CNN of an algorithm to form memories of shape, color, and other traits [18]. Thus, in this study, algorithm training was performed with transfer training, and the results of the previous training of the algorithm were used to optimize the subsequent training of the algorithm. In this study, the initial weights of YOLOv5, Faster R-CNN, and YOLOv3 networks were obtained from the pre-trained models on the COCO data set, and the obtained initial weights were used for transfer training on the cotton pollen data set (as described in Materials and Methods Section 4.1, Section 4.4 and Section 4.5). Through this method, the training time was reduced, the computer hardware requirements were reduced, and the accuracy was improved. The model parameters were initialized and adjusted according to the characteristics of the pollen detection task. The model training weight was saved every 20 epochs. When the model learning rate and loss value tended to be stable, model training was considered to be over, and the optimal weights of the three models on the verification set were saved (Figure 1).

Then, the YOLOv5, Faster R-CNN, and YOLOv3 models were tested on an untrained data set to verify the accuracy and counting performance of each model. We used two indicators to evaluate the performance of each model: (1) the mean average accuracy (mAP@0.5:0.95), i.e., the position relationship between the predicted and real values of the label box, and (2) the correlation coefficient (*R*^2^) used to evaluate the quantitative difference between the predicted value and the real value. In this study, we hoped to establish a pollen viability recognition model with high mAP and *R*^2^ values as the final tool system for plant researchers.

### 2.2. Comparison of the Accuracy of the Three Algorithms

**Figure 2:**
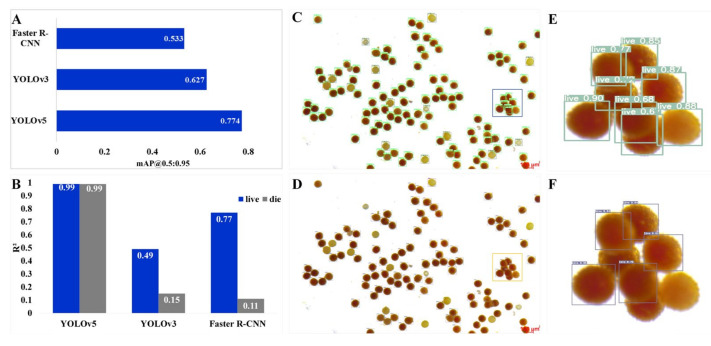
Performance evaluation of the pollen viability detection model based on deep learning. (**A**) The YOLOv5, Faster R-CNN, and YOLOv3 models were used to detect pollen in 16 images, and the position accuracy of the different models was judged by comparing the gap between the predicted frame position and the manually marked boundary box position. (**B**) Sixteen images were detected by the YOLOv5, Faster R-CNN, and YOLOv3 models, and the counting accuracy of the different models was judged by analyzing the correlation with the actual values (*R*^2^). (**C**,**D**) The visualization results of the same cotton pollen image obtained by the YOLOv5 model and YOLOv3 model, respectively. Bars: 100 µm. (**E**,**F**) An enlarged image of the box in (**C**,**D**), respectively.

mAP@0.5:0.95 is a commonly used index for evaluating the quality of deep learning models. We used the optimal weights of the three models for prediction on the same verification set and compared the accuracy of the three models for pollen viability detection tasks. The difference in the mAP@0.5:0.95 values of the three optimal weights was obvious, showing a ladder distribution (Figure 2A). The performance of the YOLOv5 model was the best (mAP@0.5:0.95 was 0.774), the mAP@0.5:0.95 of the YOLOv3 model was medium (0.627), and the mAP@0.5:0.95 of the Faster R-CNN model was the worst, at 0.533; however, this value is relatively high according to our experience (Figure 2A). In summary, the three models showed high levels of accuracy, and could be embedded within prediction systems, in which the performances of the YOLOv5 and YOLOv3 models were better than that of the Faster R-CNN model.

### 2.3. Comparison of the Counting Performance of the Three Algorithms

The accuracy of the counting of viable and nonviable pollen is critical to modeling. We used the optimal weight of the three models to predict the same test set, and compared the output with the real value. The results show that YOLOv5 was the most reliable model, with an *R*^2^ of 0.99 for both the ‘alive’ tag and the ‘dead’ tag. On the other hand, the Faster R-CNN model and YOLOv3 model were not satisfactory for the detection of ‘dead’ tags, with *R*^2^ values of only 0.15 and 0.11, respectively, while for ‘alive’ tags, the performance of the Faster R-CNN model reached a high level (*R*^2^ = 0.77), but the *R*^2^ of the YOLOv3 model was only 0.49 (Figure 2B). The results show that the optimal weights of the three models differed greatly for counting performance in pollen viability detection tasks (Figure 2B). YOLOv5 and YOLOv3 were then used to detect the same pollen staining picture, and the results are shown in Figure 2C,D. It is obvious that for pollen from the same area in the image, YOLOv5 accurately detected all the samples and marked the correct categories (Figure 2E), while YOLOv3 ignored many pollen grains (Figure 2F). In summary, both the YOLOv5 and Faster R-CNN models performed well in terms of accuracy for the recognition of ‘alive’ tags, but for the detection of ‘dead’ tags, the YOLOv5 model showed a great advantage over the other two models.

### 2.4. Transfer of the Detection Capability of PollenDetect to Pollen from Different Plants

**Figure 3:**
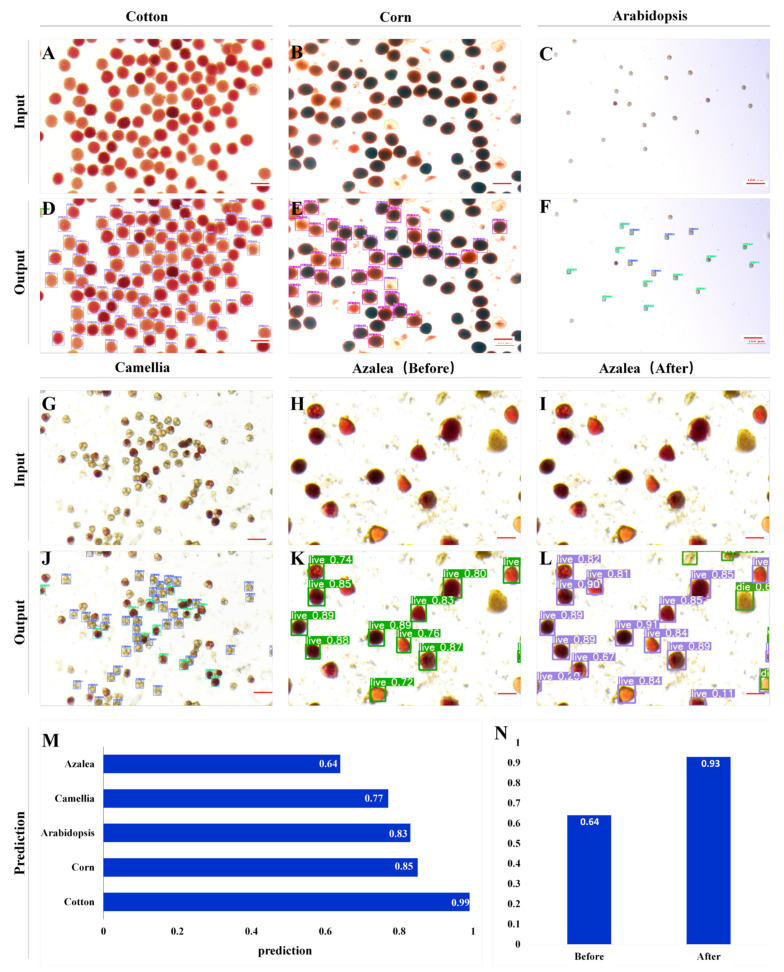
Direct extension of the trained model to the pollen of other plants. (**A**–**C**,**G**–**I**) The input image of pollen stained with TTC solution for cotton (**A**), corn (**B**), *Arabidopsis* (**C**), camellia (**G**), azalea ((**H**) before fine-tuning), and azalea ((**I**) after fine-tuning). (**D**–**F**,**J**,**K**) The training algorithm was used to visualize the results of pollen staining image detection for cotton (**D**), corn (**E**), *Arabidopsis* (**F**), camellia (**J**), and azalea ((**K**), before fine-tuning). (**L**) The training algorithm after fine-tuning was used to visualize the results of azalea pollen staining image detection. (**M**) The results of model detection were compared with those of manual labeling, and the average accuracy of model detection was calculated. (**N**) Before and after fine-tuning, the detection results for azalea were compared with those of manual labeling, and the average accuracy of model detection was calculated. (**A**–**L**) Bars: 100 µm.

As discussed above, the PollenDetect tool built on the basis of YOLOv5 was the optimal model for detecting cotton pollen viability (Figure 2). To examine the knowledge transfer capability of YOLOv5 and assess whether the developed algorithm can be used on pollen of other plants, the PollenDetect tool was used to detect the pollen viability of other plants. We chose corn (monocotyledon), *Arabidopsis* (dicotyledonous plant), camellia, and azalea (ornamental flowers), which are different from cotton in terms of pollen to some extent. Cotton pollen has spines, while the outer layer of *Arabidopsis* and corn pollen is smooth. In addition, cotton and corn pollen is globoid, while *Arabidopsis* pollen is spindle-shaped, and the pollen of ornamental flowers is tetrahedral in shape. The pollen of the four kinds of plants (corn, *Arabidopsis*, camellia, and azalea) was stained with TTC using the same experimental procedure used for the cotton pollen data set, and the pollen staining images were photographed with a camera-connected microscope. We used PollenDetect to detect the pollen in 10 images of each plant, and compared the results with the results of manual counting, to evaluate the performance of the model in terms of pollen viability detection for different plants. The detection results show that the accuracy of PollenDetect for cotton, *Arabidopsis*, corn, camellia, and azalea pollen viability was 99%, 83%, 85%, 77%, and 64%, respectively (Figure 3).

To solve the problem of low accuracy when applied to pollen viability counting of other plants, we further fine-tuned the YOLOv5 algorithm using a set of eight annotated azalea images for additional training and training for 200 epochs, and then tested 10 images. The fine-tuning improved the accuracy from 64% to 93%. For example, in the same pollen staining image of azalea, pollen that could not be detected before fine-tuning were detected after fine-tuning and correctly classified as viable or nonviable (Figure 3H,I,K,L). The above fine-tuning process took only 1 h. Thus, the knowledge transfer from cotton to other plants was successful and straightforward, as expected.

### 2.5. Detection of Cotton Pollen High-Temperature Tolerance via PollenDetect

**Figure 4:**
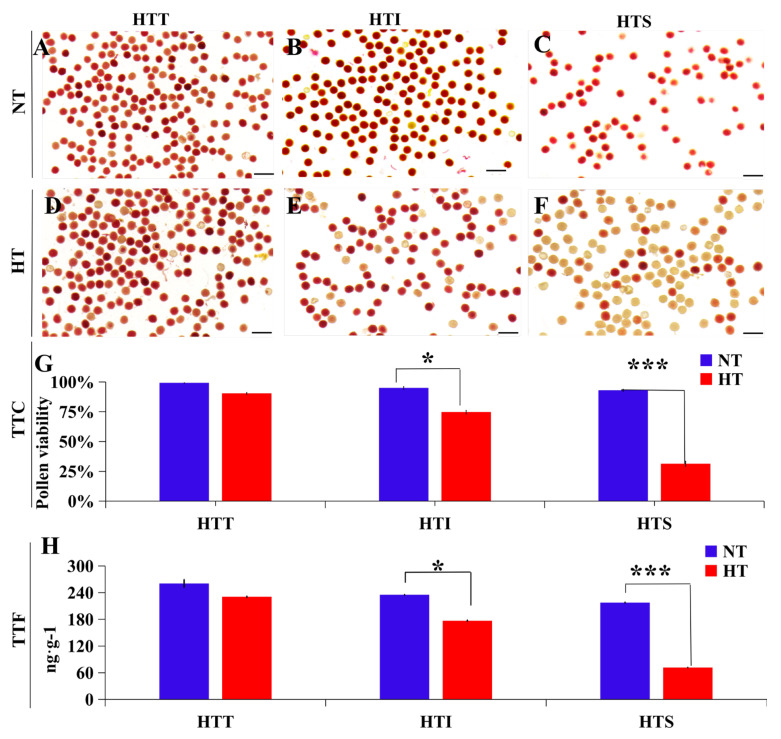
Pollen viability analysis via PollenDetect recognition of TTC staining images corresponding with the TTF quantitative analysis results. (**A**–**C**) Pollen TTC staining images of HTT (**A**), HTI (**B**), and HTS (**C**) cotton lines under NT conditions. Bars: 100 µm. (**D**–**F**) Pollen TTC staining images of HTT (**D**), HTI (**E**), and HTS (**F**) cotton lines under HT conditions. Bars: 100 µm. (**G**,**H**) TTC staining and TTF quantitative methods were used to calculate the pollen viability of cotton varieties with different HT tolerances. Values are the mean ± s.d. for three biological replicates. Asterisks indicate statistically significant differences (* *p* < 0.05; *** *p* < 0.001, Student’s *t*-test). HTT, HT-tolerant; HTI, intermediately HT-tolerant; HTS, HT-sensitive; NT, normal temperature; HT, high temperature; TTC, 2,3,5-triphenyl tetrazolium chloride; TTF, 2,3,5-triphenyltetrazolium formazan.

In our previous study, we explored the TTF quantification analysis of pollen viability in anthers under normal and HT conditions [19] (Chinese patent number: ZL201910010240.2). To confirm the accuracy of PollenDetect and the correlation between TTC staining and TTF quantification results, three cotton lines with different tolerances to HT were selected for analysis. The HT-tolerant (HTT) cotton line, intermediately HT-tolerant cotton line (HTI), and HT-sensitive cotton line (HTS) were treated under normal temperature and HT conditions. For each line, TTC staining recognized by PollenDetect and TTF quantification were used to calculate pollen viability (Figure 4). TTC pollen staining recognition by PollenDetect showed that the pollen viability of the three cotton lines decreased to varying degrees after HT treatment: the pollen viability of HTT slightly decreased (Figure 4A,D,G), that of HTI and HTS both decreased significantly, and HTS pollen viability decreased more dramatically than that of HTI (Figure 4B,C,E–G), consistent with the results of TTF quantification (Figure 4H). We believe that PollenDetect can be used to detect pollen viability and screen stress-resistant plants with high pollen viability.

## 3. Discussion

The development of a high-throughput tool is of great significance for quickly evaluating pollen viability status and then screening and locating target genes in combination with genome-wide association technology. Our study explored an efficient and accurate method based on deep learning to detect viable and nonviable pollen, which can reduce the workload of researchers and significantly increase the yield of reliable quantitative data. This shows the great advantage of using deep learning in agricultural image processing.

In a previous study, machine learning was used to detect plant pollen quality according to the grain and shape characteristics of pollen [20]. Furthermore, pollen recognition based on deep learning mostly focuses on crop pollen detection and counting [21], while in this study, PollenDetect could not only detect the number of pollen grains, but also accurately identify whether each pollen grain was viable. Because pollen exhibits a high contrast with a pure-color background, the largest indicator of whether the pollen is viable or nonviable is the difference in colors after staining. In this study, the pretraining model had excellent generalization performance in the detection of cotton pollen, and was used in training. After training three different deep learning networks (YOLOv3, YOLOv5, and Faster R-CNN), the YOLOv5 model was chosen because it had the best mAP@0.5:0.95 value (0.774) and the best recognition accuracy (99%) (Figure 2A,B), while the YOLOv3 and Faster R-CNN models had lower detection accuracies for nonviable cotton pollen (Figure 2A). The identification of viable and nonviable pollen grains is equally important, as both involve in the calculation of pollen viability rates. In addition, for many cotton pollen aggregates, probably due to their unclear boundaries and mutual occlusion, the YOLOv3 and Faster R-CNN models could not accurately detect all cotton pollen grains, while the YOLOv5 model could accurately detect occluded and overlapping targets (Figure 2C,F) due to the addition of the NMS algorithm. Therefore, special attention should be given to this phenomenon when imaging and using PollenDetect software.

In this study, we gave priority to the classification counting accuracy of the model rather than the general performance index mAP of the model. For counting performance regarding viable and nonviable cotton pollen, the accuracy of the model reached 99% (Figure 2B). We speculate that higher recognition accuracy can be achieved using a more streamlined network structure (YOLOv5s) and increasing the number of samples included in the training process; furthermore, the reasoning speed of the model can be accelerated at the same time.

When PollenDetect is directly applied to the pollen of corn and *Arabidopsis thaliana*, only some parameters needed to be fine-tuned, no retraining was needed, and the detection accuracy of the model exceeded 83% (Figure 3M). In addition, we also used pollen from ornamental flowers (camellia and azalea) to test the detection effect of PollenDetect (Figure 3G,H,J,K). The detection performance of the model did not sharply decline with respect to whether the pollen grains had spines or whether the plants were monocotyledons or dicotyledons. To achieve the same detection accuracy observed for cotton pollen, one simply needs to expand the data set, and the new data set needs to include more kinds of plant pollen and images under different light conditions. After migration training, PollenDetect can constantly learn the characteristics of pollen of different plants and improve their detection. The refinement of the pre-existing YOLOv5 models using azalea images for an extra training phase (also called fine-tuning) yielded better results in terms of count accuracy (Figure 3N). Notably, during this generalization, we only used 18 pictures for fine-tuning (training and validation), compared to 74 pictures that were used for cotton.

The use of PollenDetect on pictures of pollen from different laboratories allowed us to identify some of the limitations of the counting algorithm: (1) the light source was not uniform when the images were taken, resulting in different tones within each group of images, which affected the model’s recognition of whether the pollen was viable, and (2) regarding the cleanliness of the image background, when more impurities were mixed with the stained pollen, it was possible to identify some impurities as pollen, which affected the detection accuracy. The first point depends on the experimental setup, which could be overcome by fine-tuning our model using images from different laboratories. We are able to provide some guidance for overcoming the second issue. We provided a detailed description of handling pollen prior to taking photographs (see Section 4) to reduce the number of impurities in the pollen batch. Alternatively, a new batch of pollen training images containing impurities is recommended before the use of PollenDetect to detect unclean pollen batches [22].

In this study, we verified the effect of high temperature on cotton pollen viability [19,23,24] through PollenDetect software. We believe that changes in other environmental conditions will also cause changes in pollen activity, and researchers can use the software we provide to perform high-throughput pollen screening.

## 4. Materials and Methods

### 4.1. Plant Materials and Growth Conditions

The cotton germplasms were planted at the Breeders Base of the Institute of Economic Crops, Xinjiang Academy of Agricultural Sciences, Arvati Township, Korla (86.08° E, 41.68° N), and the cotton experimental field and greenhouse of Huazhong Agricultural University (114.35° E, 30.47° N). For cotton growth in the field, 23 °C to 32 °C during the day and 20 °C to 27 °C at night were set as normal temperature conditions (NT). The high-temperature (HT) treatment included temperatures above 35 °C during the day and above 27 °C at night. In the greenhouse, the plants were grown at 28 °C to 35 °C during the day and 20 °C to 27 °C at night as normal conditions. During HT treatment in the greenhouse, the plants were cultivated at 35 °C to 39 °C during the day and 29 °C to 31 °C at night. Single plants growing in flowerpots were maintained in the greenhouse. The soil was mixed with gravel and substrate at a 1:2 ratio.

*Arabidopsis thaliana* ecotype Columbia (*Col-0*) was grown in an incubator under long-day conditions (16 h light/8 h dark) with white fluorescent light at 20 °C and a relative humidity of 60%. Corn plants were planted in the experimental field of Huazhong Agricultural University in Wuhan, Hubei Province, China (114.35° E, 30.47° N). The row spacing of the corn was 90 cm, and the initial distance between corn plants in each row was 30 cm. The temperature conditions were the same as those for cotton planted in the experimental field of Huazhong Agricultural University. Camellia and azalea were planted in the Germplasm Resource Garden of the Forestry and Fruit Tree Research Institute, Wuhan Academy of Agricultural Sciences (114.24° E, 30.34° N).

### 4.2. TTC Staining

Under NT, anthers were collected when the pollen was fully dispersed on the day of anthesis. Anthers from five flowers of each line were collected as a biological replicate. The anthers were placed in 0.1 Mol L-1 2,3,5-triphenyl tetrazolium chloride (TTC) solution (8 g TTC was added to 1 L phosphate buffer at 0.1 M and pH = 7; China patent number: ZL201910010240.2) for staining for 60 min. HT samples were collected after 3 d of HT treatment with the same processes. After TTC staining, pollen viability was observed with a Nikon SM25 stereomicroscope (Japan), and images were collected by a charge-coupled device (CCD). The viable pollen became red after staining, while the nonviable pollen was gray or unstained. A pipette was used to absorb 1 mL of the stained pollen and place it on a glass slide to make a slice. Three slices were made for each flower, and three evenly distributed visual fields were randomly selected for photography. To generate enough pollen samples for deep learning model training at the same developmental stage, all stained pollen grains were imaged (Figure S1). A total of 74 usable images of stained pollen were collected, and a total of 8232 stained pollen samples were obtained, including 6068 viable cotton pollen samples and 2164 nonviable pollen samples. Pollen grains from all plant species were processed the same way.

### 4.3. TTF Quantitation

Anthers were collected in 2 mL tubes with TTC solution (less than 1 mL), weighed, stained for 60 min, and then ground for 70 s at 60 Hz using a TissueLyser-192 (Shanghai Jingxin, Shanghai). Next, 700 µL ethyl acetate was added to the tubes and mixed well by vortexing. After centrifugation for 5 min at 12,000 rpm/min, 200 µL supernatant was transferred into a microtiter plate. Absorbance at 485 nm was measured using a multimode plate reader (Perkinelmer EnSpire, Turku, Finland), and the concentration of 2,3,5-triphenyl tetrazolium formazan (TTF, ng/g fresh weight) was calculated. Each line had three biological replicates, each replicate included five anthers, and each anther sample had three technological replicates.

### 4.4. Image Annotation

The purpose of our study was to determine whether we could directly examine cotton pollen staining images to identify whether each pollen grain is viable through a deep learning model without any other tools or systems. According to our observation of the samples, the color difference of stained and unstained pollen was obvious in the RGB channel, and could be used to distinguish whether pollen is viable or nonviable. The viable cotton pollen was dark red and reddish brown after TTC staining, while the nonviable cotton pollen was light yellow or gray (Figure S2); thus, we distinguished the viable and nonviable cotton pollen by color characteristics. In this experiment, Labellmg (version 1.8.4) software was used to label the cotton pollen staining images, and a minimum outer rectangle was applied for each pollen grain visible in the image. According to the pollen color after staining, viable pollen was labeled ‘alive’, while nonviable pollen was labeled ‘dead’, so that each pollen grain had an independent tag as ground truth that could be used in the deep learning model (Figure S3).

### 4.5. Data Preprocessing

The labeled cotton pollen images were randomly divided into a training set and a verification set at a ratio of 8:2 (Figure S4). To prevent overprediction in certain species due to sample imbalance, the ratio of viable to nonviable cotton pollen in the data set was set as 3:1 for the tested species, and the weights of categories with fewer samples were increased to avoid errors in model training caused by the dominant number of sample categories in the data set.

### 4.6. YOLOv5 Detection Algorithm

**Figure 5:**
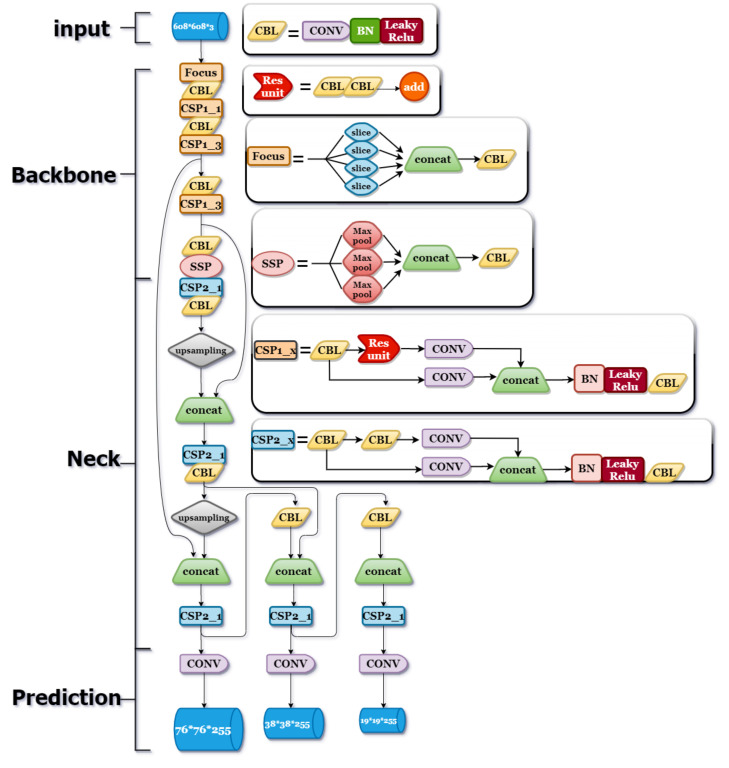
YOLOv5 network structure. The YOLOv5 network includes the input side, backbone side, neck side, and prediction side [25]. The input side is mainly responsible for mosaic data enhancement and adaptive anchor frame calculation. The backbone side has a focus structure and CSP structure. The neck side is made up of the main part of the FPN + PAN structure. The prediction side uses GIOU_LOSS to output image detection results. CSP, cross-stage partial; SSP, spatial pyramid pooling; BN, batch normalization; CONV, convolution; GIOU_LOSS, generalized intersection over union loss.

YOLOv5, released in June 2020, is the latest target detection framework in the YOLO series. It runs on a Tesla P100 and has an inference time of 0.07 s per image, which is half the time of YOLOv4. YOLOv5 has higher recognition accuracy and recognition speed than the previous generations. The YOLOv5 algorithm is especially suitable for portable devices that cannot carry a large GPU. Since the pollen size is relatively fixed, there is no need to use a large detection frame. Thus, the YOLOv5 deep learning algorithm was used for pollen recognition training, and the structure contained four aspects, i.e., input, backbone, neck, and prediction, which are shown in Figure 5.

#### 4.6.1. Input Side

The YOLOv5 input side mainly includes mosaic data enhancement, adaptive anchor frame calculation, and adaptive image scaling. Mosaic data enhancement is one of the algorithms unique to YOLOv5 [26]. The proportion of small targets is usually slightly lower in daily visual recognition data sets. To meet the need for small target recognition, four images were stitched together with random scaling, cropping, and arrangement. This approach makes the algorithm more sensitive to small targets, such as cotton pollen, and enriches their data sets.

An adaptive anchor frame calculation was performed by setting different initial anchor frame lengths and widths. The algorithm generates possible initial prediction frames based on the initial anchor frames, compares them with the calibrated ground truth, calculates the differences between them, and then updates them in reverse, iterating the network parameters. The initial anchor frames set by YOLOv5 in this study are (116, 90, 156, 198, 373, 326) (30, 61, 62, 45, 59, 119) (10, 13, 16, 30, 23).

Adaptive image scaling fills differently sized images with differently sized black edges at both ends so that they become standard-sized images. For this algorithm, the Letterbox function was used to fill both sides of an image with black edges to reduce computation and improve speed.

#### 4.6.2. Backbone

The backbone is a CNN that aggregates and forms image features from different image minutiae, including the focus structure and cross-stage partial (CSP) structure [25].

Focus structure is a major innovation in YOLOv5 that does not appear in other versions of the YOLO series. The focus structure mainly performs an image slicing operation, as shown in Figure S5. The slicing operation preserves image features while reducing image size by extracting image feature values, which speeds up the inference of the algorithm. For example, in our deep learning algorithm, after a pollen image of 608 × 608 × 3 pixels was input into the focus structure, a feature mean average precision (mAP) of 304 × 304 × 12 pixels was output, and then after one convolution operation with 32 convolution kernels, a feature mAP of 304 × 304 × 32 pixels was output.

The main function of the CSP structure is to reduce the computation and memory cost while ensuring accuracy. The convolution kernel size of each CSP module at the backbone side is 3 × 3 with a step size of 2, which serves as a downsampling function by convolving the original image. A total of five CSP modules were used in this experimental algorithm. The size of the feature mAP changed from 608 × 608 to 19 × 19 after five iterations. The CSP structure was incorporated into both the backbone and neck ends of this algorithm.

#### 4.6.3. Neck

A neck is a series of network layers that mix image features and pass them to a prediction layer. The neck side in this experiment included a path aggregation network, feature pyramid network (FPN), and pixel aggregation network (PAN) structure [27], in addition to the CSP structure mentioned above. As shown in Figure S5, the FPN structure delivers strong semantic features by downsampling from top to bottom, followed by two PAN structures that deliver strong localization features by downsampling from bottom to top, aggregating features from different backbone layers for different detection layer information to extract the most feature information. This not only preserves the image features, but also reduces the size of the data set that needs to be processed, speeds up the algorithm’s reasoning, and increases its recognition accuracy.

#### 4.6.4. Prediction

The prediction side extracts the image features to generate bounding boxes and predict their categories. In this experiment, GIoU_Loss was used as the bounding box regression loss function, and weighted non-maximum suppression (NMS) was used to exclude redundant candidate boxes [28,29]. Since cotton pollen grain overlap with and obscure each other, using only the NMS algorithm resulted in the inability to accurately identify adjacent pollen grains, causing a few pollen images with the highest confidence to be retained. Therefore, we used the soft-NMS algorithm [30]. This method can effectively prevent the omission of a certain target or repeated detection of a certain target when a single target position overlaps.

### 4.7. YOLOv3 Detection Algorithm Design

YOLOv3 is an end-to-end one-stage target detection model. The basic idea of YOLOv3 algorithm prediction is as follows: first, the feature of the input image is extracted through the feature extraction network, and the feature mAP of a specific size is obtained and output. Next, the input image is divided into 13 × 13 network elements, and if the central coordinates of a category in the real box fall within a network element, then the network element predicts the category. Each category has a fixed number of bounding boxes, and there are three bounding boxes within each network unit in YOLOv3. Logical regression is used to determine the regression boxes and then to predict a category. The YOLOv3 model of this experiment adopted the Darknet-53 feature extraction network, which is a 53-layer full convolution network structure that pursues the accuracy of detection while maintaining a certain detection speed.

### 4.8. Faster R-CNN Detection Algorithm Design

Faster R-CNN is a two-stage target detection model. The specific idea of Faster R-CNN model detection is as follows: first, the CNN network is used to extract the features of the input image, and the feature mAP of the image is obtained. Next, the candidate box is obtained from the feature mAP through the RPN network, the candidate box is classified (to determine whether it belongs to the foreground or the background), and the position relationship between the candidate box and the labeled real box is adjusted by the regression algorithm in the foreground to make them similar. This step is the most important difference between the two-stage detection model and the single-stage detection model. The candidate box obtained in the previous step is sent to multiple classifiers for classification to determine whether the selected area of the candidate box belongs to a specific category.

### 4.9. YOLOv5 Model Refinement

After the 17th layer of the network structure, the original network structure is modified to continue the upsampling operation to expand the feature map, and at the 20th layer, the acquired 160 × 160 and the 2nd layer of the backbone network are used to obtain a larger feature map for small target detection. In the detection layer, a small target detection layer is added to use a total of four layers of structure (22, 25, 28, 31) for detection.

### 4.10. Model Evaluation Metrics

The main evaluation metrics applied in this study were mAP@0.5:0.95 and accuracy. The formula is expressed as follows:

$$P\left(Precision\right)=\frac{T_{P}}{P_{N}}$$

$$R\left(Recall\right)=\frac{T_{P}}{T_{N}}$$

$$\mathrm{accuracy}=\frac{T_{P}+T_{N}}{T_{P}+F_{P}+T_{N}+F_{N}}$$

$$AP=\int_{0}^{1}P\left(R\right)dR$$

In the above formula, *T_P_* is the number of instances that are actually positive instances and classified by the classifier as positive instances; *F_P_* is the number of instances that are actually negative instances and classified by the classifier as positive instances; *F_N_* is the number of instances that are actually positive instances and classified by the classifier as negative instances; *T_N_* is the number of instances that are actually negative instances and classified by the classifier as negative instances; mAP is the average of the average precision (AP) of all categories. mAP@0.5:0.95 is the process of increasing intersection over union (IoU) from 0.5 to 0.95 with steps of 0.05. The mAP corresponding to each IoU is added to obtain the average value of mAP in this process.

## 5. Conclusions

In this study, we developed and validated software, PollenDetect, as a high-throughput and efficient tool for pollen viability detection (viable or nonviable) and counting. For the cotton pollen data set, the mAP@0.5:0.95 of the model was 0.774, the accuracy was 99%, and the correlation *R*^2^ with manual calibration results was 0.99. This is the first study to apply a deep learning target detection model to the high-throughput analysis of pollen viability. This model can replace manual counting or quantification to determine the viability of large quantities of pollen. The obtained pollen viability data can be used to identify the target genes that affect the response of pollen to high temperature and other stresses. For the convenience of other users, PollenDetect was embedded into a user-friendly interface (Figure S6) and uploaded to GitHub (https://github.com/Tanzhihao1998/Identification-of-pollen-activity.git, accessed on 1 April 2022), where it can be downloaded and freely used by all internet users. The software allows users to integrate pollen staining images into the same folder for rapid batch detection. The test results are output for further statistical analysis. Our output contains not only the total number of pollen grains, but also the number of viable and nonviable pollen grains in the same image.

### Limitations and Future Work

We demonstrated the possibility of applying the model to the pollen of other plants (*Arabidopsis*, corn, and ornamental flowers). Thus, fine-tuning some of the parameters and training the model can allow it to be applied in pollen viability detection modules for other plants. However, the current precision of pollen detection for other crops needs to be further improved. In view of the transferability of knowledge, we will continue to add pollen detection modules of other plants to this model in the future. In addition to this, other phenotypes of pollen should also be detected, e.g., volume size of pollen, circumference of pollen. We intend build an integrated model for the detection of multiple phenotypes of pollen in the future.

## Data Availability

Details and source code of the PollenDetect model used in this study are openly available at https://github.com/Tanzhihao1998/Identification-of-pollen-activity.git/ (accessed on 1 April 2022).

## Supplementary Materials

The following supporting information can be downloaded at: https://www.mdpi.com/article/10.3390/ijms232113469/s1. Figure S1: Scheme of how the pollen image was obtained using a microscope. The images were taken, generating overlapping areas to obtain a macro image without blemishes in these areas. Figure S2: Close-up of cotton pollen. (A,B): Inviable cotton pollen grains. (C,D): Viable cotton pollen grains. Bars in (A–D): 10 μm. Figure S3: Graphic environment of the manual labeling software used for the classification of the pollen. In the central part, it can be seen how the pollen grains have been labeled by an expert and classified according to their status. Figure S4: Data set composition. The data set contained 74 pollen images, and the ratio of the training set to the validation set was 8:2. Figure S5: Focus schematic and FPN + PAN structure. (A): The original image inputted by the model was 4 ∗ 4 ∗ 3, the eigenvalues of the image were extracted by the focus structure, and the 2 ∗ 2 ∗ 12 image was output. This structure can effectively reduce the size of the image, speed up the reasoning of the model, and maintain the original features of the image. (B): Pollen images output by the FPN+PAN structure with 19 ∗ 19, 38 ∗ 38, and 76 ∗ 76 feature mAPs corresponding to different sizes of anchor boxes were used for prediction. FPN: feature pyramid network; mAP: mean average precision; PAN: path aggregation network. Figure S6: PollenDetect software interface. The software can select a single image or a group of images for pollen viability detection, and the test results will be counted and output to the text box for further statistics and analysis.

## Abbreviations

CNNs	convolutional neural networks
R-CNN	region-CNN
CSP	cross-stage partial
mAP	mean average precision
FPN	feature pyramid network
PAN	pixel aggregation network
NMS	non-maximum suppression
HT	high temperature
HTT	HT-tolerant
HTI	intermediately HT-tolerant
HTS	HT-sensitive
