# Supplementary material for: PollenDetect: An Open-Source Pollen Viability Status Recognition System Based on Deep Learning Neural Networks
Source: Int J Mol Sci. 2022 Nov 3;23(21):13469. doi: 10.3390/ijms232113469 (PMC9653958; doi:10.3390/ijms232113469)
Supplement: Supplementary file 1 [file ijms-23-13469-s001.zip › ijms-1943402-supplementary.pdf]

Supplementary Materials

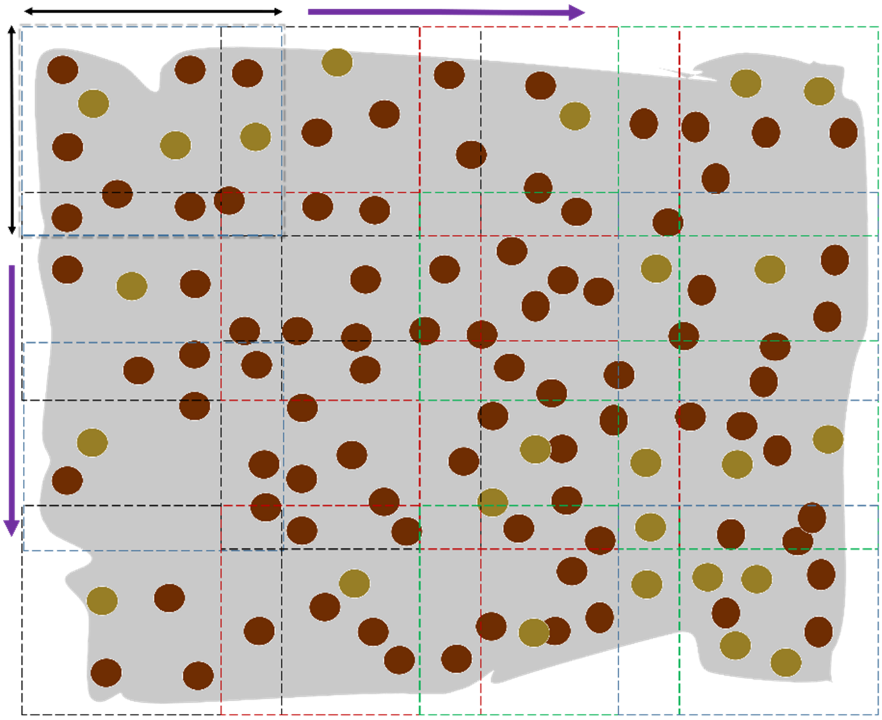

Figure S1. Scheme of how the pollen image was obtained using a microscope. The images were taken, generating overlapping areas to obtain a macro image without blemishes in these areas.

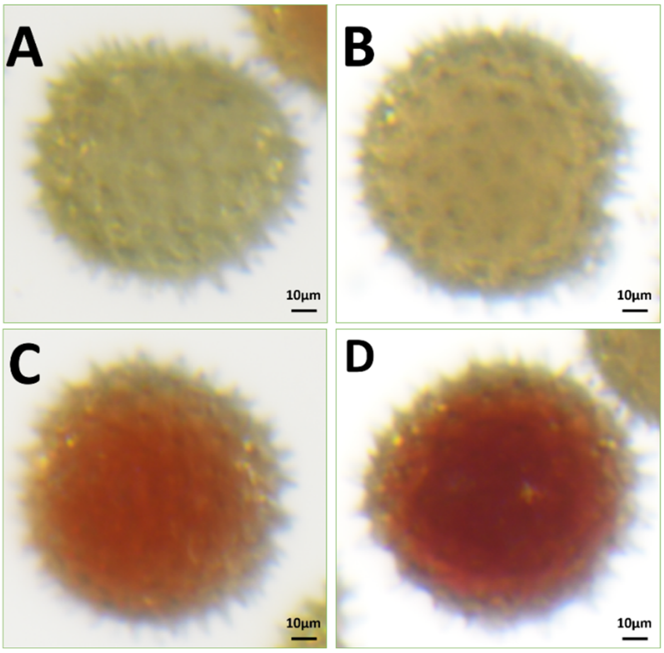

Figure S2. Close-up of cotton pollen. (A,B): Inviable cotton pollen grains. **(C,D)**: Viable cotton pollen grains. Bars in (**A-D**): 10 µm.

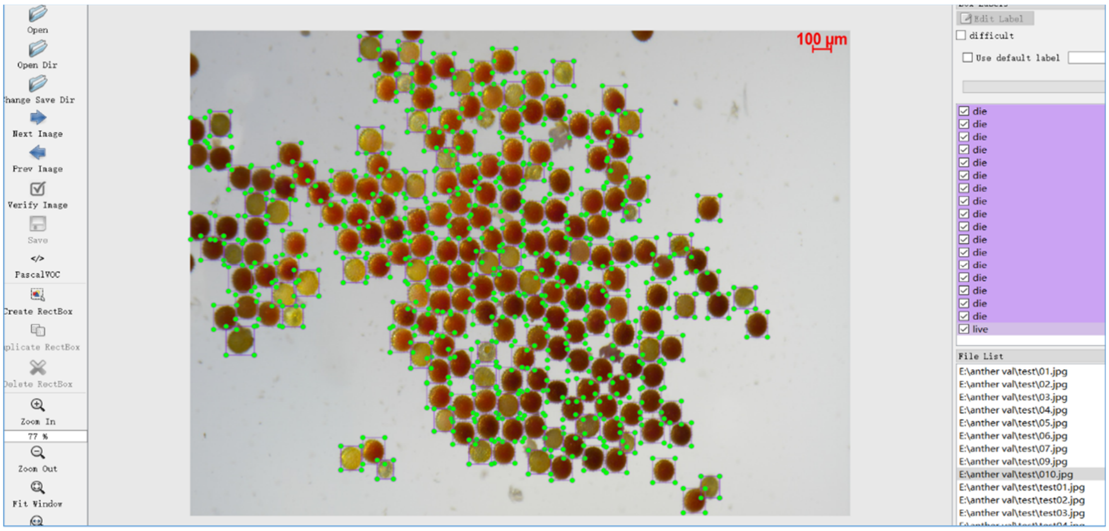

Figure S3. Graphic environment of the manual labeling software used for the classification of the pollen. In the central part, it can be seen how the pollen grains have been labeled by an expert and classified according to their status.

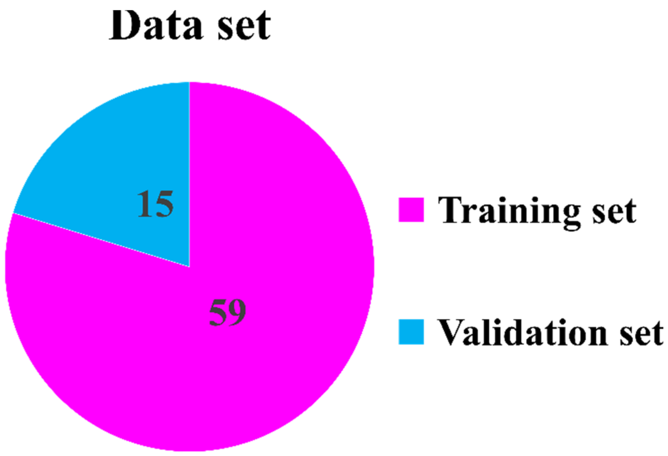

Figure S4. Data set composition. The data set contained 74 pollen images, and the ratio of the training set to the validation set was 8:2.

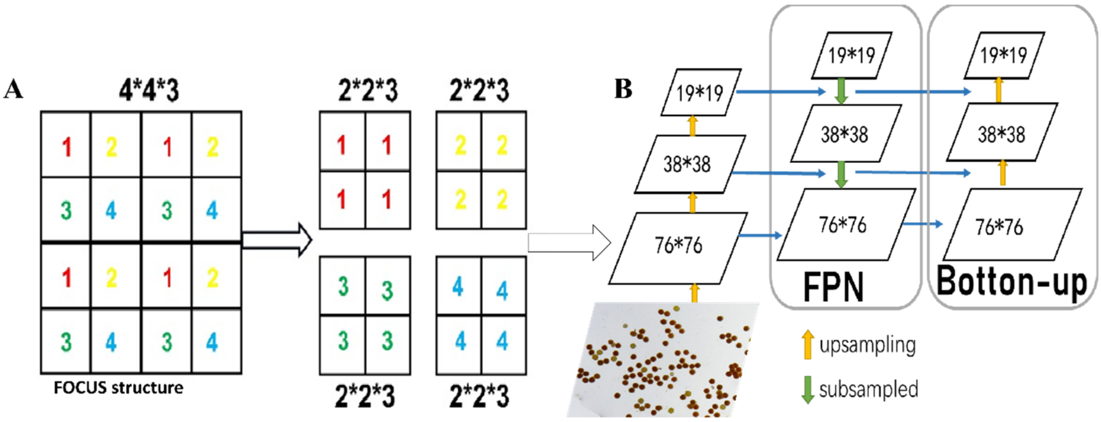

Figure S5. Focus schematic and FPN+PAN structure. (**A**): The original image inputted by the model was 4*4*3, the eigenvalues of the image were extracted by the focus structure, and the 2*2*12 image was output. This structure can effectively reduce the size of the image, speed up the reasoning of the model, and maintain the original features of the image. (**B**): Pollen images output by the FPN+PAN structure with 19*19, 38*38, and 76*76 feature mAPs corresponding to different sizes of anchor boxes were used for prediction. FPN: feature pyramid network; mAP: mean average precision; PAN: path aggregation network.

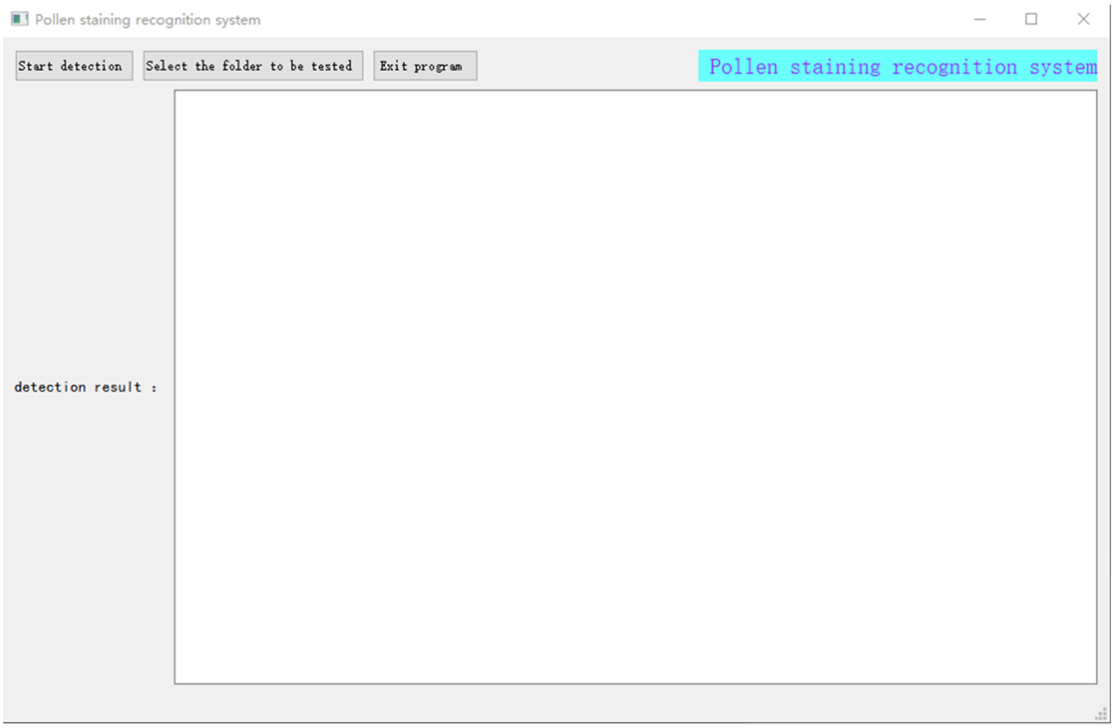

Figure S6. PollenDetect software interface. The software can select a single image or a group of images for pollen viability detection, and the test results will be counted and output to the text box for further statistics and analysis.
